# AuNP/Chitosan Nanocomposites Synthesized through Plasma Induced Liquid Chemistry and Their Applications in Photothermal Induced Bacteria Eradication

**DOI:** 10.3390/pharmaceutics14102147

**Published:** 2022-10-10

**Authors:** Zhijun Guo, Dan Sun, Xian Zhou, Huan Xu, Yizhou Huang, Chenglin Chu, Baolong Shen

**Affiliations:** 1 School of Materials Science and Engineering, Southeast University, Nanjing 211189, China; 2 School of Materials and Physics, China University of Mining and Technology, Xuzhou 221116, China; 3 School of Mechanical and Aerospace Engineering, Queen’s University, Belfast BT9 5AH, UK; 4 Laboratory of Stem Cell and Tissue Engineering, Orthopedic Research Institute, State Key Laboratory of Biotherapy and Cancer Center, West China Hospital, Sichuan University and Collaborative Innovation Center of Biotherapy, Chengdu 610041, China

**Keywords:** atmospheric pressure micro-plasma, plasma induced liquid chemistry, gold nanoparticles, chitosan, nanocomposites, photothermal conversion, antibacterial

## Abstract

In this work, a facile direct current atmospheric pressure micro-plasma (APM) technology was deployed for the synthesis of functional gold nanoparticle/chitosan (AuNP/CS) nanocomposites for the first time. Different experimental parameters, such as metal salt precursor concentration and chitosan viscosity, have been investigated to understand their effects on the resulting nanocomposite structures and properties. The nanocomposites were fully characterized using a wide range of material characterization techniques such as UV–vis, transmission electron microscope (TEM), Fourier transform infrared (FTIR) spectra and X-ray photoelectron spectroscopy (XPS) analyses. Potential reaction pathways have been proposed for the nanocomposite synthesis process. Finally, potential of the synthesized nanocomposites towards photothermal conversion and bacteria eradiation applications has been demonstrated. The results show that APM is a facile, rapid and versatile technique for the synthesis of AuNP/CS functional nanocomposites. Through this work, a more in-depth understanding of the multi-phase system (consisting of gas, plasma, liquid and solid) has been established and such understanding could shine a light on the future design and fabrication of new functional nanocomposites deploying the APM technique.

## 1. Introduction

Gold nanoparticles (AuNPs), amongst various functional nanomaterials, have attracted great attention in the biomedical field due to their promising physicochemical properties [1]. For instance, when surface modified (e.g., by thiol or amine groups), AuNPs can bind with therapeutic agents to achieve targeted drug delivery [2]. Additionally, AuNPs exhibit localized Surface Plasmon Resonance (LSPR) effect and can convert Near-Infrared (NIR) laser light energy into heat [3]. Such unique features have enabled their applications in biosensing [4], cancer hyperthermia [5] and eradication of drug resistant bacteria [6]. Despite their promising applications, the health risk associated with free AuNPs often presents a concern on the final fate of AuNPs in vivo [7]. To minimize the uncontrolled release of AuNPs, polymer-based nanocomposites incorporating AuNPs have been developed as an alternative solution [8,9]. Chitosan (CS), a biocompatible and biodegradable natural polymer, is commonly used in a wide range of biomedical applications such as prevention of biofilm formation [10], tissue engineering [11] and wound dressings [12]. Combining CS with AuNPs is shown to improve the chemical, physical and biological properties of AuNPs [10], as CS and their derivates can enhance the stability/dispersion of the AuNPs through surface functioning via hydrophilic moieties (-NH_2_, -NH^3+^, -OH, etc.) [13]. 

To date, researchers have explored various approaches for the preparation of AuNP/CS nanocomposites for functional applications such as electrochemical immunosensors [14], antimicrobial coatings [15,16], drug delivery devices [17], tissue repair scaffolds [18] and cancer treatments [19]. More details of these can be found in a recent review [20]. Early preparation methods for AuNP/polymer nanocomposites usually involve physical mixing of pre-synthesized AuNPs (e.g., those obtained from traditional Turkevich method [21,22]) with polymer solution or in situ chemical reduction of gold salt (e.g., HAuCl_4_) in polymer solutions using sodium borohydride or sodium citrate as the reduction agents [23]. In recent studies, Lu et al. synthesized AuNP-CS through reflux and boiling method. During this process, AuNPs were directly reduced and stabilized by CS, forming a positively charged AuNP-CS core. Subsequently, pH-responsive charge-reversible polymers were deposited onto the Au-CS through electrostatic interaction [24]. Labala et al. prepared AuNP-CS by direct reduction of HAuCl_4_ using CS at 100 °C, poly(N-vinyl pyrrolidone) was added as a stabilizer [25]. Shaabani et al. synthesized layer-by-layer CS capped AuNP by direct reduction of HAuCl_4_ by CS under stirring and reflux, followed by centrifugation [26]. Other non-wet chemistry-based synthesis approaches such as γ-irradiation [27], microwave [23], UV [20,28] and thermal irradiations [29,30,31] have also been explored. Unfortunately, most of the above-mentioned methods require complicated multi-step synthesis, elevated temperatures, and/or the use of potentially hazardous/harsh chemicals or irradiation. Several researchers also explored the use of plant-based non-toxic reducing agents to induce in situ formation of AuNPs inside CS matrix [23]. However, such processes usually require high temperature and long processing time.

Apart from exploring different synthetic approaches, the effects of CS molecular characteristics on AuNP formation have also been investigated recently by several researchers. For instance, Ibrahim et al. reported that increasing CS molecular weight (MW) can lead to increased AuNP size in microwave synthesized AuNP/CS composites. Other researchers, when synthesizing AuNP/CS composites using in situ thermal methods, found that the MW of CS did not have an effect on AuNP size. Rather, deacetylation [32,33] and concentration [32] of CS played a more significant role. The review of literature suggests that structure of the AuNP/CS nanocomposites (and the interaction between CS and AuNPs) is dependent on the synthetic method.

In recent years, a facile nanocomposite synthesis strategy based on the use of atmospheric pressure micro-plasma (APM)-liquid interaction has emerged [33,34]. When treating aqueous solutions containing gold salts by APM, the plasma-induced liquid chemistry can produce abundant energetic/reactive species (e.g., solvated electrons, OH•, H•, O•, and H_2_O_2_), which lead to the in situ nucleation and growth of AuNPs. Through APM-liquid interactions, a wide range of AuNP containing nanocomposites have also been successfully synthesized, including AuNP/carbon nanotubes [5,35], AuNP/graphene oxide [4], AuNP/poly(3,4-ethylene dioxy-thiophene) polystyrene sulfonate and AuAgNP/poly (vinyl alcohol) hydrogel [36] nanocomposites. Comparing to other one-pot synthesis methods, APM is a “greener” synthesis approach. By inducing plasma activated liquid chemistry, APM creates highly dispersed/stable metal NPs within the matrix material without the need of toxic reducing chemicals or stabilizing agents. Such synthesis process involves multiphase interaction (involving gas, plasma, solid and liquid), the reaction pathway of which is highly complicated and would follow entirely different mechanisms as compared to the traditional wet chemistry approaches. So far, no study has investigated the feasibility of using APM-induced liquid chemistry for the in situ synthesis of AuNP/CS nanocomposites. The effects of metal salt concentration and the polymer viscosity within such unique and complicated reaction system remain unexplored.

In this work, we report for the first time one-pot synthesis of AuNP/CS nanocomposites through APM-induced liquid chemistry. The effects of the gold salt concentration and the viscosity of CS on the resulting structures/properties of AuNP/CS nanocomposites have been investigated in detail. Formation mechanism of AuNP/CS nanocomposites under the plasma-induced liquid chemistry has been discussed and an in-depth understanding into the interfacial interactions between AuNPs and CS during the synthesis process has been established. We also processed the nanocomposite samples into thin films, to demonstrate their potential in photothermal conversion and bacteria eradication applications.

## 2. Materials and Methods

### 2.1. Materials

Chitosan (CS, deacetylation > 95%) with low/medium/high (l-CS, m-CS, h-CS) average viscosity (50–100 mPa·s, 200–400 mPa·s, and >400 mPa·s, respectively) were purchased from Rhawn Reagent Co., Ltd., Shanghai, China. Acetic acid and chloroauric acid trihydrate (HAuCl_4_·3H_2_O, > 99.9%) were purchased from Aladdin Reagent Co., Ltd., Shanghai, China. Deionized water (18.2 MΩ·cm^−1^) was produced by a Millipore Milli-Q machine.

### 2.2. Preparation of AuNP/CS Nanocomposites

As-purchased CS (l-CS, m-CS, h-CS) were dissolved in appropriate amount of 2% (*v*/*v*) acetic acid under room temperature to make 2 wt% CS aqueous solutions. Appropriate amount of 10 mM HAuCl_4_ solution was introduced into the CS/acetic acid aqueous solutions to obtain HAuCl_4_/CS mixtures with 1% CS and different HAuCl_4_ concentrations. m-CS as was chosen as the matrix polymer to investigate the effect of gold salt (HAuCl_4_) concentration (ranged from 2.5 µM to 2.0 mM) on the resulting AuNP size within the composite. 0.5 mM HAuCl_4_ was used to investigate the effect of different CS viscosity on the resulting AuNP size within the nanocomposites. Table 1 summarizes the samples prepared in this study.

**Scheme 1:**
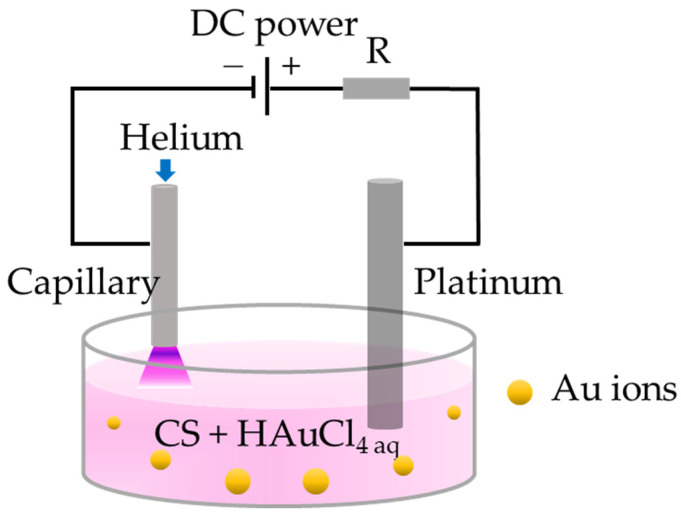
Diagram of the APM set-up used in this work.

After 30 min of stirring, the HAuCl_4_/CS mixtures were subjected to APM treatment. Scheme 1 shows the APM set-up used in the present work. The anode (platinum rod) was immersed in the HAuCl_4_/CS aqueous mixtures, while the cathode (stainless steel capillary with an inner diameter of 250 µm) was positioned approximately 1 mm above the mixture surface. Helium (He) gas was supplied through the capillary and the flow rate was regulated to 50 sccm using a mass flow controller (CS200-A, Sevenstar, Beijing, China) and a read-out unit (D08-2F, Sevenstar, China). The plasma can be ignited at the gas/liquid interface at a voltage of ~4 kV (DC power supply: LAS-10KV-12Ma-P, Boer Co., Jiangsu, China). Afterwards, the APM processing current was adjusted to 5 mA to sustain the plasma and the HAuCl_4_/CS solutions were gently stirred using a magnetic stirrer throughout the treatment (10 min). For comparison, pure CS solutions with different viscosity were also treated under the same APM condition.

AuNP/CS composite films were prepared by casting 9 mL of each as-synthesized composite liquid sample onto a 5 cm × 9 cm Teflon slide. The samples were then oven-dried at 60 °C overnight (until no further weight loss). Control film sample (pure CS and APM treated CS) were also prepared for comparison.

### 2.3. Characterization

UV−Vis spectroscopy (UV-2600, Shimadzu, Kyoto, Japan) was used to analyze the optical absorption of the resulting AuNP/CS nanocomposite solution. The sample was diluted 10 times using DI water, drop-casted on to TEM grid and dried completely before transmission electron microscope (TEM) analysis (Tecnai G2 F20/30, FEI, Hillsboro, OR, USA). The TEM images were analyzed by “FUJI” software based on >100 particles for each sample. The viscosity of samples was tested using a sensing viscometer (DV-2 pro, Brookfield, Middleboro, MA, USA). Zetasizer nanoparticle analyzer (Zetasizer Nano ZSE, Malvern, UK) was used for particle size and surface charge when appropriate. All data were averaged over three measurements to ensure repeatability. X-ray photoelectron spectroscopy (XPS) of AuNP/CS samples was conducted using a XPS system (ESCALAB 250Xi, Thermo Fisher Scientific, Waltham, MA, USA). The survey spectra were obtained with a pass energy of 150 eV, while the core level spectra were obtained using a pass energy of 20 eV. All spectra were calibrated with the C1s core peak at the binding energy of 284.8 eV and the results were analyzed using open-source software CasaXPS. Fourier transform infrared (FTIR) spectra analysis was carried out using a FT-IR Spectrometer (Spectrum 3, Perkin-Elmer, Akron, OH, USA). The thermal properties of composite film samples were investigated by differential scanning calorimetry (DSC) analysis in an N_2_ atmosphere at a heating rate of 10 °C min^−1^ using Netzsch DSC 404 F3 (Netzsch, Weimar, Germany). The photothermal conversion effect of each composite film sample (5 mm diameter discs) was tested using an NIR CO_2_ laser (wavelength = 808 nm, power density = 0.8 W cm^−2^). The temperature of the samples was monitored in situ using an IR thermal camera (FOTRIC 340) during laser irradiation.

The swelling ratio of the film samples was tested in phosphate buffer solution (PBS) at 37 °C following gravimetric method [36]:

Swelling ration (%) = [(Ws − W_d_)/W_d_] × 100 (1)

where the W_s_ and W_d_ represent the weight of the swollen film and the dried film, respectively.

### 2.4. Antibacterial Testing

Gram-negative *Escherichia coli* (*E. coli* ATCC 11303) and gram-positive *Staphylococcus aureus* (*S. aureus* ATCC 6538) were used to evaluate the antibacterial properties of the AuNP/CS nanocomposites. Composite film discs (5 mm) were placed in a 96-well cell culture plate and incubated with 100 μL (1 × 10^5^ CFU/mL) bacterial solution under NIR laser irradiation (0.8 W cm^−2^, 808 nm) for 0, 30, and 180 s, respectively. Blank well was used as a control. After NIR laser irradiation, the culture was further incubated at 37 °C for 4 h, the co-cultured bacterial suspensions were diluted with 15 mL of PBS, and 50 μL of the bacterial suspensions was removed and spread onto Mueller-Hinton agar plates and incubated at 37 °C overnight. The bacterial colonies were counted. Tests were repeated five times for repeatability.

### 2.5. Statistical Analysis

Data were expressed as mean ± standard deviation and analyzed using the SPSS 16.0 software (SPSS Inc., Chicago, IL, USA). Significant differences among groups were assessed using the one-way analysis of variance (ANOVA), followed by the least significant difference multiple comparison test. Statistical high significance and very high significance were defined as ** *p* < 0.01 and *** *p* < 0.001, respectively.

## 3. Results and Discussion

### 3.1. Physical Properties of AuNP/CS Nanocomposites

**Figure 1:**
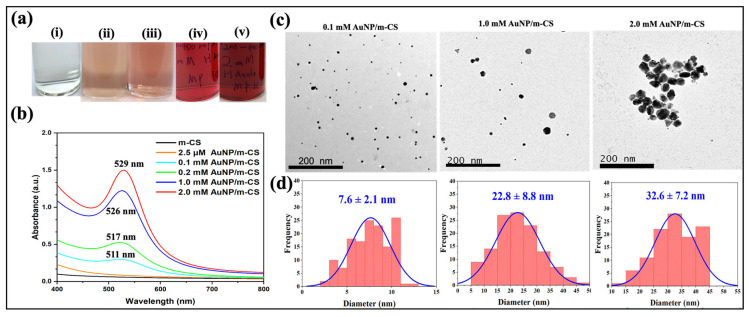
(**a**) Optical images of the as-prepared m-CS (i), 0.1 mM AuNP/m-CS (ii), 0.2 mM AuNP/m-CS (iii), 1.0 mM AuNP/m-CS (iv), and 2.0 mM AuNP/m-CS (v) nanocomposites. (**b**) UV-vis spectra of the as-prepared m-CS and AuNP/m-CS nanocomposites. (**c**) Typical TEM images and (**d**) corresponding AuNP size distribution of 0.1 mM AuNP/m-CS, 1.0 mM AuNP/m-CS, and 2.0 mM AuNP/m-CS nanocomposites.

During the APM treatment, all sample solutions showed immediate color change (Figure 1a), compared to the pure m-CS solution (Figure 1a). The color of the plasma treated samples ranged from pale pink to mahogany with increasing HAuCl_4_ concentration. The optical property of each solution sample was analyzed using UV-vis spectroscopy (Figure 1b). In the absence of HAuCl_4_, the APM treatment had no significant influence on the absorption spectrum of CS. For AuNP/m-CS nanocomposite solutions, the concentration of HAuCl_4_ caused a red shift of the absorption resonant peak from 511 nm (0.1 mM) to 529 nm (2.0 mM). The characteristic peak wavelength is in agreement with the LSPR band of AuNPs [37] and the red shift of LSPR is indicative of the growing AuNP size and the particle anisotropy [37,38]. The intensity of LSPR, on the other hand, is related to the concentration of AuNP in the solution, which gradually increased with the increasing HAuCl_4_ concentration. Although the light scattering caused by the nanoparticles could to certain extent affect the overall detected optical signal, this is considered insignificant and is not the main focus of this study, hence it will not be further investigated here. The morphology and size of AuNPs formed within the APM treated AuNP/m-CS were further investigated by TEM. Representative TEM images in Figure 1c illustrate the typical AuNP morphologies resulted from different HAuCl_4_ concentration. At lower HAuCl_4_ concentrations, the AuNPs (dark particles) are well-dispersed in the CS matrix and are mostly in spherical shapes. The particle size distribution analysis (Figure 1d) suggests that the average AuNP size increases with increasing HAuCl_4_ concentration, consistent with the red shift of the LSPR peak found in the UV-vis analysis. The result is similar to previous studies where the size of APM synthesized metal nanoparticles increases with metal salt concentration [34,36]. On the other hand, with increasing HAuCl_4_ concentration, AuNPs also start to show signs of anisotropy (e.g., triangular, hexagonal, rods, etc.). This is in line with the literature where the metal salt precursor concentration is reported to have an impact on the AuNP growth kinetics [39]. The UV-vis and TEM results suggest that the HAuCl_4_ concentration plays a significant role on the AuNP size, morphology and the size distribution within the AuNP/CS nanocomposites. 

**Figure 2:**
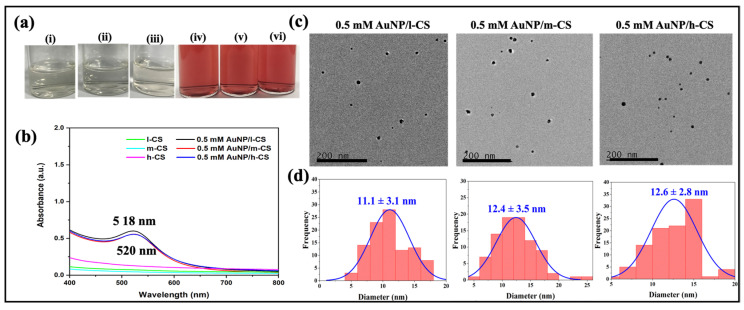
(**a**) Optical images of l-CS (i), m-CS (ii), h-CS (iii), 0.5 mM AuNP/l-CS (iv), 0.5 mM AuNP/m-CS (v), and 0.5 mM AuNP/h-CS (vi). (**b**) UV-vis spectra of untreated (l, m, h)-CS and APM-treated 0.5 mM AuNP/(l, m, h)-CS nanocomposites. (**c**) TEM images and (**d**) the corresponding AuNP size distribution of 0.5 mM AuNP/l-CS, 0.5 mM AuNP/m-CS, and 0.5 mM AuNP/h-CS nanocomposites.

To investigate the effect of CS viscosity on the morphology and size of the synthesized AuNPs, CS with different viscosities, namely l-CS (50–100 mPa·s), m-CS (200–400 mPa·s) and h-CS (>400 mPa·s) were deployed. All APM-treated samples exhibit consistent color (ruby red) (Figure 2a). Figure 2b shows that the LSPR for APM-treated HAuCl_4_/(l-CS, m-CS, h-CS) was independent of the CS viscosity. This result suggests that the AuNP size and concentration were not influenced by the initial CS viscosity. This can be further supported by the TEM images in Figure 2c, where all APM-treated samples contain well-dispersed spherical AuNPs within the CS with similar particle size distribution (11.1 ± 3.1, 12.4 ± 3.5, and 12.6 ± 2.8 nm, respectively) (Figure 2d). 

The fact that the AuNP size is independent on the initial CS viscosity is in contrast to the results reported in [33] where AuNP synthesized by in situ thermal method showed a decreasing trend with increasing CS viscosity. This is because the APM treatment has significantly altered the CS viscosity during the treatment (Figure S1). It is reported that the plasma-induced liquid chemistry can produce abundant energetic/reactive species (such as solvated electrons, radicals, OH•, H•, and O•, etc.) [33,35]. It is widely accepted that OH• can induce the cleavage of β-1-4 glycosidic linkages and the oxygenation of d-glucopyranose rings within the CS chains, leading to effective CS chain scission [40,41], hence reducing the polymer viscosity. All APM treated samples showed similar viscosity regardless of the initial CS concentration. As a result, their impact on the AuNP growth kinetics is similar.

The chemical structural properties of the treated samples were studied by FTIR (Figure S3). The broadband ranging from 3200 to 3500 cm^−1^ can be attributed to the –OH and –NH_2_ group stretching vibrations within the CS molecular chains. The peaks around 2927, 2848, 1334, and 1250 cm^−1^ can be correlated to the CH_2_ bending within pyranose rings [42]. The peaks at around 1639 cm^−1^ are due to the vibrations of C=O (amide I) of O-C-NHR, while the peak at around 1540 cm^−1^ is the NH bending (amide II) (NH_2_) peak. The band at 1152 and 1064 cm^−1^ were attributed to the C-O-C bend of saccharide structures. The band at 1408 cm^−1^ is due to CH_3_ wagging [43] and the peak at 1338 cm^−1^ can be attributed to the C-C stretching of the glucosamine group of CS [44]. It is noticed that all peak intensities of APM-treated AuNP/CS were weakened as compared to pure CS, indicating the coverage of CS signal by AuNPs.

**Figure 3:**
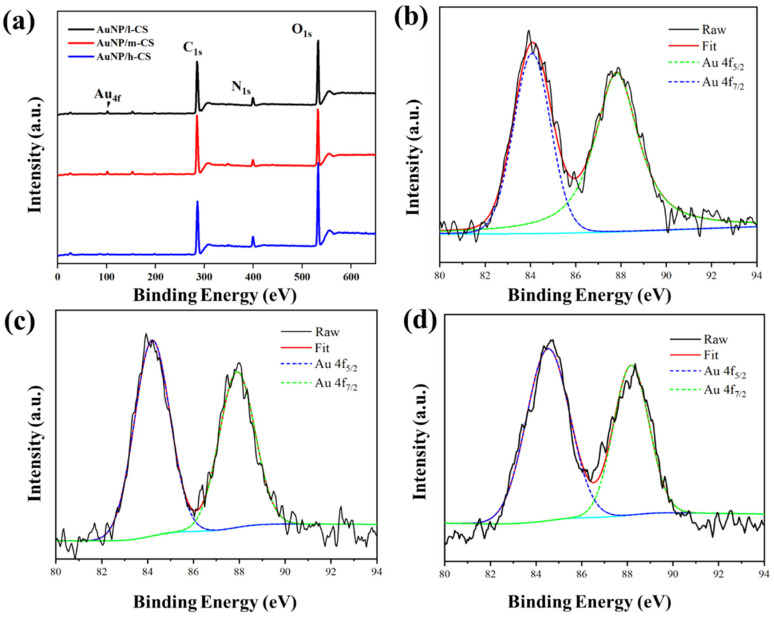
(**a**) Survey XPS spectra of 0.5 mM AuNP/l-CS, 0.5 mM AuNP/m-CS, and 0.5 mM AuNP/h-CS nanocomposites. (**b**–**d**) Au 4f core XPS spectrum of 0.5 mM AuNP/l-CS, 0.5 mM AuNP/m-CS, and 0.5 mM AuNP/h-CS nanocomposites, respectively.

XPS analysis of APM-treated AuNP/CS nanocomposites containing (l, m, h)-CS was carried out to confirm the atomic state of Au element. The survey scans of the three samples all present distinguishing Au peaks (Figure 3a). The core Au 4f of each sample (Figure 3b–d) can be well fitted into two doublet centering at binding energy (BE) of 84.0 and 87.7 eV, respectively. The two element states of Au 4f are consistent with the profiles of reduced metallic Au^0^ [45], suggesting Au^3+^ ions have been reduced to form Au^0^ NPs by APM treatment. The detailed BE and atomic fraction of all fitted elements are listed in Table S1. No absorbance at 313 nm (characteristic LSPR of Au^3+^) has been detected in APM-treated samples (Figure S2), indicating the depletion of Au^3+^ (i.e., 100% reduction) during the APM process [46]. The results suggest our APM technique is an efficient technique in producing well dispersed AuNPs in comparison to the traditional thermal synthesis approach. As with the latter method, 100% Au^3+^ → Au^0^ reduction could not be achieved and the as-synthesized AuNPs were more aggregated and anisotropic in morphology [32].

### 3.2. AuNP/CS Nanocomposite Formation Mechanisms

**Scheme 2:**
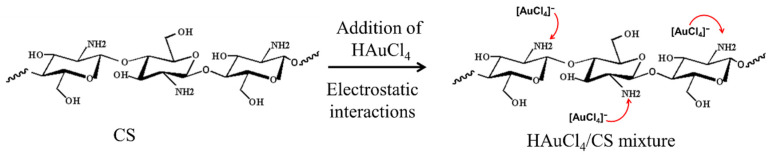
A schematic showing the electrostatic interactions between [AuCl_4_]^−^ and the amide groups on CS.

The formation of AuNPs within the CS network is influenced by both the plasma induced liquid chemistry and the presence of CS. For HAuCl_4_ aqueous solution (without CS), two absorption bands are present at 220 and 280 nm (Figure S2-green curve), which correspond to the $p_{\pi }5d_{x^{2}-y^{2}}$ and $p_{\sigma }5d_{x^{2}-y^{2}}$ ligand−metal transition modes in the [AuCl_4_]^−^ complex structures, respectively [47]. For HAuCl_4_/CS mixture solution, the corresponding absorption peaks showed decreased intensity and were red shifted, indicating part of [AuCl_4_]^−^ were anchored onto the CS (Figure S2-red curve) [48]. This transition can be attributed to the interaction between [AuCl_4_]^−^ complex and the CS -NH_2_ group through ion exchanging reaction as -NH_2_ becomes ionized into -NH^−^ and H^+^ in water [49]. In contrast, no ion exchange should in principle take place between -OH and [AuCl_4_]^−^ because −OH cannot be ionized in water [34] (Scheme 2). This interaction is also evidenced by the UV-vis spectra shown in Figure S2, where the [AuCl_4_]^−^ band (220 nm and 280 nm) in the UV-spectrum became weakened in the 0.2 mM HAuCl_4_/CS mixture samples. Note that, depending on the initial HAuCl_4_ concentration, any free/unbound [AuCl_4_]^−^ in the bulk solution can also participate in the reduction reaction.

**Scheme 3:**
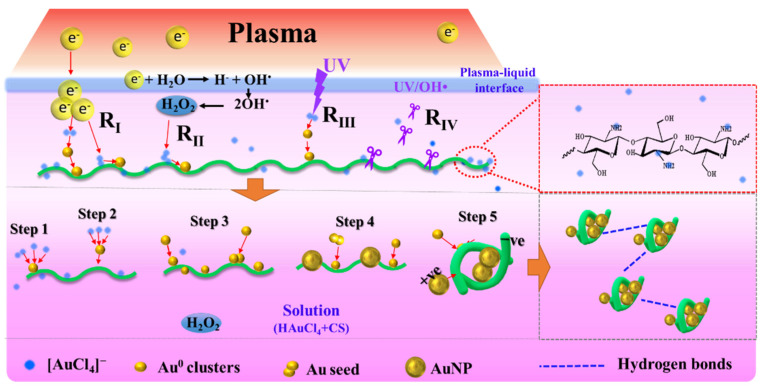
Schematic showing the potential reaction mechanisms involved in the APM synthesis of AuNP/CS nanocomposites.

The formation mechanism of AuNP/CS nanocomposites as a result of APM treatment has been illustrated in Scheme 3. Solvated electrons (RI) and H_2_O_2_ (RII) are believed to be the two main species that trigger the initial reduction of [AuCl_4_]^−^ [50,51,52]. When the sample was subject to APM treatment, the [AuCl_4_]^−^ anchored on the CS chain and the unbound free [AuCl_4_]^−^ ions in the bulk solution were reduced into free Au^0^ atoms through both RI and RII reaction pathways, see Equations (2) and (3).

(2) $${\mathrm{Au}}^{3+}+3e^{-}\to {\mathrm{Au}}^{0}$$

(3) $${\mathrm{Au}}^{3+}+{\mathrm{OH}}^{-}+3H_{2}O_{2}\to {\mathrm{Au}}^{0}+3H_{2}O+3{\mathrm{HO}}_{2}$$

In addition, the APM process is also accompanied by UV radiation [53,54]. The UV light generated in the plasma could also partly contribute to the AuNP formation (Scheme 3 RIII) following Equation (4) [55]. It is widely accepted that the OH• and UV produced by the APM-liquid interaction can lead to the scission of CS chains (Scheme 3 RIV) [40,41].

(4) $${\mathrm{Au}}^{3+}+3e^{-}\overset{\mathrm{hv}}{\to }{\mathrm{Au}}^{0}$$

**Figure 4:**
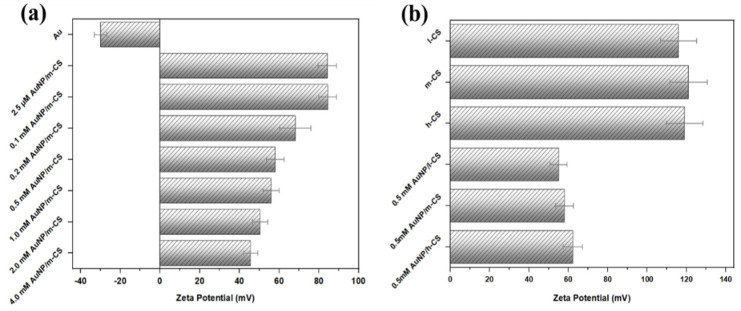
Zeta potential of samples under investigation: (**a**) Au, 2.5 µM AuNP/m-CS, 0.1 mM AuNP/m-CS, 0.2 mM AuNP/m-CS, 0.5 mM AuNP/m-CS, 1.0 mM AuNP/m-CS, 2.0 mM AuNP/m-CS, 4.0 mM AuNP/m-CS, (**b**) l-CS, m-CS, h-CS, 0.5 mM AuNP/l-CS, 0.5 mM AuNP/m-CS, 0.5 mM AuNP/h-CS.

It is worth pointing out that the reducing power of short-lived solvated electrons is usually constrained at the plasma-liquid interfaces, while H_2_O_2_ molecules can diffuse into the bulk solution and enable the reduction of the remaining [AuCl_4_]^−^ via Equation (3) [40]. Although the dominant reduction mechanism is not easy to quantify, we hypothesize the reaction pathway RI is mainly responsible for the initial AuNP nucleation and the subsequent AuNP growth is via aggregation and coalescence near the plasma-liquid interface [36]. This is because the solvated electrons travel much faster than H_2_O_2_ [52]. AuNP reduced by H_2_O_2_ would be mainly driven by the coalescence and surface growth in the bulk solution [56]. In the presence of CS, the formation of the first Au^0^ clusters can take place on the Au–N–C bonding sites on the CS surface (Step 1). Depending on the HAuCl_4_ concentration/CS ratio, this process is also possible for unbound free [AuCl_4_]^−^ in the bulk solution (Step 2). The as-formed Au^0^ clusters further coalesce with other clusters to form stable Au seed (Step 3). In the event where residual Au ions are still present in the solution after step 3, the ions will be attracted towards the seed AuNPs due to their surface electric double layer. The H_2_O_2_ within the bulk liquid will further reduce these ions into Au^0^ as part of the continuous surface growth process (Step 4) [36,57,58]. The AuNPs formed in the bulk solution were negatively charged (zeta potential: ~30 mV, see Figure 4) and they could interact electrostatically with the positively charged CS (zeta potential: ~120 mV, see Figure 4) to form AuNP/CS complex (Step 5). Within the solution, the Au seed growth will continue until the HAuCl_4_ supply is depleted. For instance, in the 2.5 μM AuNP/m-CS sample, the further growth of AuNP after the initial formation of Au seeds was not possible, due to the rapid depletion of [AuCl_4_]^−^ ions at low HAuCl_4_ concentration. Whereas for 0.1 to 2 mM AuNP/CS nanocomposites, there is a more abundant supply of [AuCl_4_]^−^ in the solution to sustain the growth of AuNPs until they reach their stability. For the same initial HAuCl_4_ concentration, as-synthesized AuNPs are similar in size and morphology, regardless of the CS viscosity (Figure 2).

### 3.3. Thermal and Swelling Properties of AuNP/CS Nanocomposite Film

**Figure 5:**
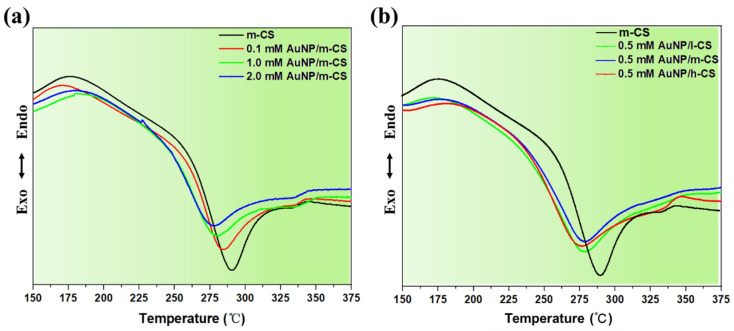
(**a**) DSC curves of m-CS, 0.1 mM AuNP/m-CS, 1.0 mM AuNP/m-CS, and 2.0 mM AuNP/m-CS films. (**b**) DSC curves of m-CS, 0.5 mM AuNP/l-CS, 0.5 mM AuNP/m-CS, and 0.5 mM AuNP/h-CS films.

The thermal properties of typical composite film samples were analyzed by DSC and the results are shown in Figure 5. The DSC thermograms of all samples exhibit a broad endothermic peak (first stage) ranging from 150 to 200 °C, which is due to the dehydration process of water constrained in the sample via hydrogen bonds [33,59]. The exothermic stage (second stage) starting from 275 °C corresponds to the decomposition of the CS polymer chains. In Figure 5a, the lower thermal decomposition temperature of AuNP/m-CS nanocomposites (275 °C) comparing to pure m-CS (290 °C) maybe due to the presence of AuNP within CS network. It is reported that AuNP has a catalytic competency over the polymer decomposition [60]. For AuNP/CS synthesized from CS of different viscosities, the endothermic peak showed more intense peak at 171, 179 and 184 °C for l-CS, m-CS and h-CS of CS. This may be due to the greater water content in the higher viscosity CS requires greater thermal absorption. Figure 5b shows that there is no variation in the exothermic peak among the three composite film samples, suggesting initial CS viscosity has no influence over the decomposition behavior of the APM synthesized composites.

**Figure 6:**
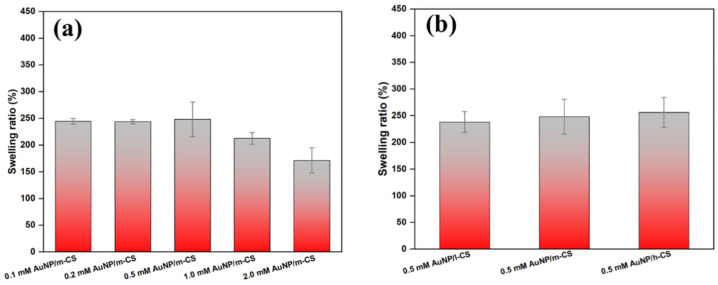
Swelling ration of typical Au/CS composite film samples in PBS. (**a**) Swelling ration of 0.1 mM AuNP/m-CS, 0.2 mM AuNP/m-CS, 0.5 mM AuNP/m-CS, 1.0 mM AuNP/m-CS, and 2.0 mM AuNP/m-CS films. (**b**) Swelling ration of the 0.5 mM AuNP/l-CS, 0.5 mM AuNP/m-CS, and 0.5 mM AuNP/h-CS films.

The water uptake ability of the nanocomposites is key to their applications particularly in the biomedical field [61]. The swelling behaviors of APM-treated CS and AuNP/CS films were evaluated and their swelling ratios are shown in Figure 6. Pure CS films dissolved completely in PBS after 24 h, whereas APM-treated l-CS, m-CS and h-CS remained stable in PBS (Figure S4). This is because the APM process has altered the molecular structures of CS, leading to a more robust cross-linked network consisting of shorter CS molecular chains [5]. This has in turn enhanced the stability of CS and prevented its dissolution in PBS. Figure S5 shows that there is no statistical difference in the viscosity of APM-treated l-CS, m-CS and h-CS, suggesting the APM treatment has led to CS solutions with similar polymer molecular structures. Compared to APM-treated pure CS, the swelling ratio of AuNP/CS nanocomposites (with HAuCl_4_ concentration > 0.5 mM) decreases with increasing initial HAuCl_4_ concentration (Figure 6a). This may be due to the greater AuNP contents within the polymer network allows less room for holding water [62]. The swelling ratio of AuNP/CS nanocomposites synthesized from different initial CS viscosities was shown in Figure 6b and no significant difference was found between the three groups, confirming the independence of the composite swelling ratio on the initial CS viscosity.

### 3.4. Photothermal Conversion of Anti-Bacterial Properties of AuNP/CS Nanocomposite Film

**Figure 7:**
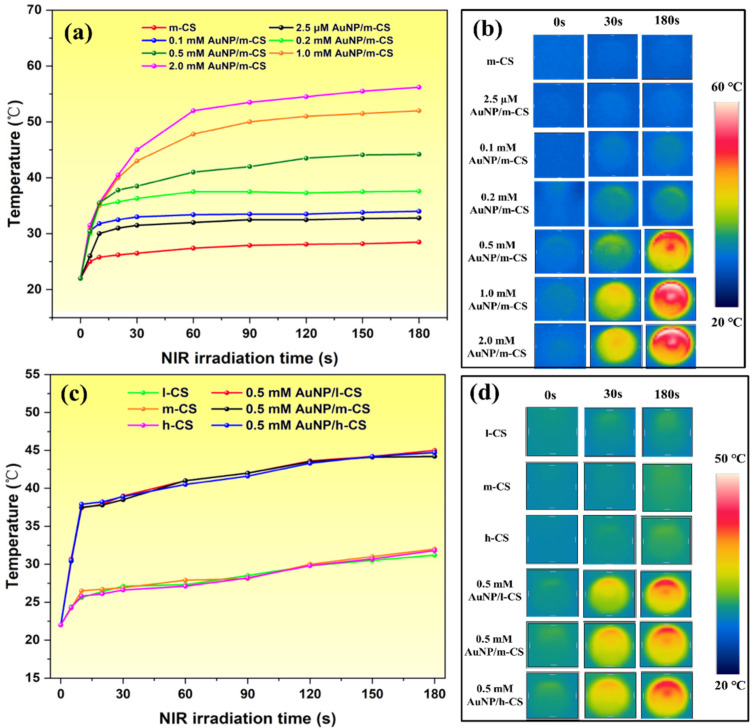
Temperature profile of AuNP/CS nanocomposites under NIR laser irradiation (power density = 0.8 W cm^−2^). (**a**) Effect of different HAuCl_4_ concentrations on the photothermal conversion efficiency of AuNP/CS nanocomposites and (**b**) their correlating IR thermal images at different time intervals; (**c**) Effect of initial CS viscosity on the photothermal conversion efficiency of AuNP/CS nanocomposites and (**d**) their correlating IR thermal images at different time intervals.

The photothermal conversion efficiency of our nanocomposite film was assessed under irradiation of 808 nm CO_2_ laser (Figure 7). All samples showed drastic temperature rise in the first 30 s, after which the temperatures curves plateaued. Results show that the sample synthesized from a greater HAuCl_4_ concentration exhibits a higher photothermal conversion efficiency (Figure 7a,b). The trend of temperature rise follows 2.0 mM AuNP/m-CS > 1.0 mM AuNP/m-CS> 0.5 mM AuNP/m-CS > 0.1 mM AuNP/m-CS > 2.5 μM AuNP/m-CS. The trend is in line with the increasing AuNP size as indicated by the UV-vis data, suggesting the particle size plays an important role in the photothermal effect. The sample of 2.0 mM AuNP/m-CS (laser power density = 0.8 W cm^−2^) exceeded 45 °C in 30 s, reaching the therapeutic window for photothermal bacteria killing [63] and cancer hyperthermia [11]. In the literature, Jo et al. created an AuNSw core-shell cancer therapy system consisting of a glycol CS core and small AuNP shell [64]. However, the photothermal conversion efficiency of such system is relatively low—it took 7 min for their samples to reach 55.5 °C under 1W/cm^2^ NIR laser irradiation. Salem et al. also developed nanogold-loaded CS nanocomposites for drug release and chemo-photothermal cancer therapy applications [65]. Yet their best sample only reached the therapeutic temperature window after ~10 min of irradiation using a 532 nm laser (200 mW).

Literature suggests that the photothermal conversion efficiencies of AuNPs can be influenced by the particle size, shape and concentration [66]. Potential formation of assemblies or aggregates during the sample preparation could also lead to red shift of the surface plasmon absorption maximum to the NIR region [67]. On the other hand, past studies report anisotropic AuNPs with multiple sharp spikes have increased number of localized plasmonic modes and could result in stronger light-heat conversion efficacy [68]. As such, the faster temperature rise profiles seen in our 2.0 mM AuNP/m-CS could be the results of growing AuNP size/concentration as well as the increasing particle anisotropy within the sample. Figure 7c,d confirms that the initial CS viscosity does not have significant impact on the resulting composite photothermal conversion efficiency. This is because the AMP- induced liquid chemistry has broken down the CS molecular chains in all samples, leading to CS solutions with the same viscosity. As such, the presence of CS can no longer affect the AuNP particle formation kinetics, and hence the resulting AuNP size/concentration and morphology are independent of the initial CS viscosity. 

**Figure 8:**
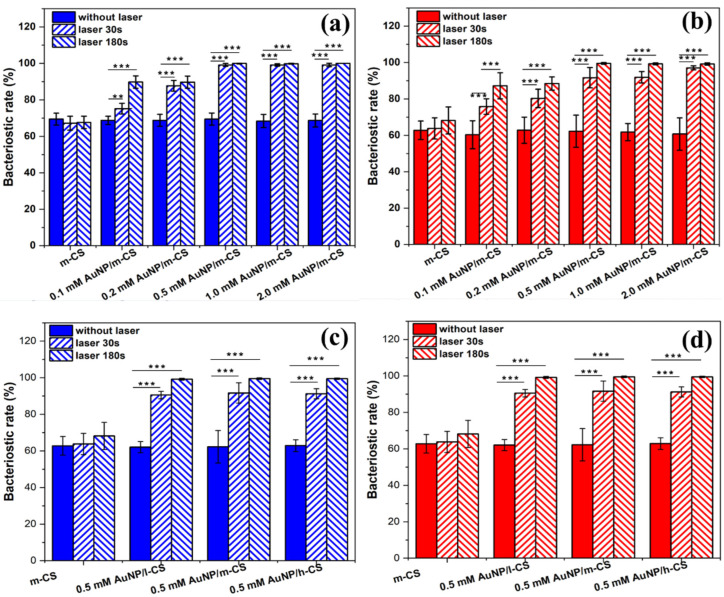
(**a**,**c**): Bacteriostatic rate of the samples against *E. coli*; (**b**,**d**): Bacteriostatic rate of the samples against *S. aureus*, ** *p* < 0.01 and *** *p* < 0.001 vs corresponding group without laser.

The strong photothermal effect of our AuNP/CS nanocomposites have been demonstrated for bacterial eradication applications. As shown in Figure 8, the bacteriostatic rate (BR) of as-prepared CS is ~65% against *E. coli* and ~60% against *S. aureus*. This is due to the inherit anti-microbial properties of CS [69]. The antimicrobial property of CS is proposed as a result of the entrance of CS molecules into bacterial cell membrane, which leads to their interactions with DNA and thus the damage of bacteria [70]. Upon 30 s of NIR laser irradiation (power density = 0.8 W cm^−2^), the BR of AuNP/CS nanocomposites dramatically increased with increasing initial HAuCl_4_ concentration, and the BR can reach as high as ~99% (Figure 8a,b). The trend of BR (i.e., increasing with initial HAuCL_4_ concentration) is consistent with that of the temperature rise profile shown by Figure 7a. For samples synthesized from greater HAuCl_4_ concentration, the resulting AuNP particle size, concentration and morphology favor the light-to-heat conversion. Samples made from higher HAuCl_4_ concentration will reach the temperature threshold sooner and photothermal damage of the bacteria cell membrane occurs as a result of thermal disintegration [71].

Comparing Figure 8a,b, our samples demonstrated slighter higher antibacterial efficiency towards *E. coli* as compared to *S. aureus* This is because *S. aureus* is surrounded by a thicker cell wall, whereas the *E. coli* cell wall comprises of a thin layer of negatively charged lipopolysaccharide [24], making it more susceptible to thermal damage. Results show that our sample demonstrate much greater bacteria killing efficiency when compared to the thiol chitosan-wrapped gold nano-shell samples developed by Manivasagan et al. In their study, the total eradication of *E. coli* was only achieved after 5 min of 808 nm laser irradiation (at a power density of 0.95 W cm^−2^) [72]. 

We further investigate the effect of initial CS viscosity on the BR efficiency of our composite film samples. Without NIR laser irradiation, 0.5 mM AuNP/l-CS, 0.5 mM AuNP/m-CS, and 0.5 mM AuNP/h-CS exhibited a similar BR towards *E. coli* (~65%) and *S. aureus* (~60%) (Figure 8c,d). With the application of NIR laser, near total eradication against *E. coli* can be achieved within 30 s, and that for *S. aureus* can be achieved within 180 s, regardless of the initial CS viscosity. Results confirm that APM-synthesized AuNP/CS nanocomposites can be successfully deployed for killing pathogenic bacteria, and the killing efficiency is directly influenced by the AuNP characteristics (size, concentration and morphology) within the composite sample. This is directed related to the initial HAuCl4 concentration, but is independent of the initial CS viscosity.

## 4. Conclusions

In this study, we have successfully demonstrated a facile approach for the synthesis of AuNP loaded CS nanocomposites deploying atmospheric pressure micro-plasma. The complicated interaction within the multiphase phase reaction system and the associated reaction pathways have been elucidated in detail. An in-depth understanding on how synthetic parameters such as HAuCl_4_ concentration and the initial polymer viscosity can affect the resulting nanocomposite structures and properties has been established for the first time. Our APM-synthesized AuNP/CS nanocomposites demonstrated excellent photothermal conversion efficiencies and anti-microbial properties under NIR laser irradiation. Such properties are highly desirable for potential applications such as antibacterial coatings and cancer hyperthermia. Although our APM approach is currently limited to bench scale synthesis, in future, the technology has the potential to be scaled up for rapid synthesis of wide range of functional nanocomposites. In future work, our study would also hugely benefit from in vitro and in vivo evaluation (such as animal studies), to warrant the potential of our composites in practical biomedical applications.

## Figures and Tables

**Table 1:** Nomenclatures of samples and their preparation methods.

Sample Acronym	CS Viscosity	HAuCl_4_ Concentration	APM
l-CS	50–100 mPa·s	none	no
m-CS	200–400 mPa·s	none	no
h-CS	>400 mPa·s	none	no
2.5 µM AuNP/m-CS	200–400 mPa·s	2.5 µM	yes
0.1 mM AuNP/m-CS	200–400 mPa·s	0.1 mM	yes
0.2 mM AuNP/m-CS	200–400 mPa·s	0.2 mM	yes
1.0 mM AuNP/m-CS	200–400 mPa·s	1.0 mM	yes
2.0 mM AuNP/m-CS	200–400 mPa·s	1.0 mM	yes
0.5 mM AuNP/l-CS	50–100 mPa·s	0.5 mM	yes
0.5 mM AuNP/m-CS	200–400 mPa·s	0.5 mM	yes
0.5 mM AuNP/h-CS	>400 mPa·s	0.5 mM	yes

## Data Availability

Not applicable.

## Supplementary Materials

The following supporting information can be downloaded at: https://www.mdpi.com/article/10.3390/pharmaceutics14102147/s1, Figure S1: The viscosities of samples under investigation, Figure S2: UV-vis spectra of 0.2 mM HAuCl_4_/m-CS mixture and untreated 0.2 mM HAuCl_4_ in water, Figure S3: FTIR spectra of (a) untreated m-CS and APM-treated 0.1 mM AuNP/m-CS, 0.2 mM AuNP/m-CS, 1.0 mM AuNP/m-CS, and 2.0 mM AuNP/m-CS groups. (b) FTIR spectra of untreated (l, m, h)-CS and APM-treated 0.5 mM AuNP/(l, m, h)-CS groups, Figure S4: Image of sample films after immersed in PBS for 24 h at 37 °C, Figure S5: Swelling ratio of AMP-treated l-CS, m-CS, and h-CS, Figure S6: Typical low magnification TEM images of 0.5 mM AuNP/l-CS, 0.5 mM AuNP/m-CS, and 0.5 mM AuNP/h-CS nanocomposites. Table S1. Au 4f peak information for 0.5 mM AuNP/l-CS, 0.5 mM AuNP/m-CS, and 0.5 mM AuNP/h-CS nanocomposites.
