# Supplementary material for: AuNP/Chitosan Nanocomposites Synthesized through Plasma Induced Liquid Chemistry and Their Applications in Photothermal Induced Bacteria Eradication
Source: Pharmaceutics. 2022 Oct 10;14(10):2147. doi: 10.3390/pharmaceutics14102147 (PMC9611015; doi:10.3390/pharmaceutics14102147)
Supplement: Supplementary file 1 [file pharmaceutics-14-02147-s001.zip › pharmaceutics-1908571-supplementary.pdf]

# AuNP/Chitosan Nanocomposites Synthesized through Plasma Induced Liquid Chemistry and Their Applications in Photothermal Induced Bacteria Eradication

**Zhijun Guo [1,2], Dan Sun [3], Xian Zhou [2], Huan Xu [2], Yizhou Huang [4,*], Chenglin Chu [1,*] and Baolong Shen [1]**

[1] School of Materials Science and Engineering, Southeast University, Nanjing 2211189, China
[2] School of Materials and Physics, China University of Mining and Technology, Xuzhou 221116, China
[3] School of Mechanical and Aerospace Engineering, Queen's University, Belfast BT9 5AH, UK
[4] Laboratory of Stem Cell and Tissue Engineering, Orthopedic Research Institute, State Key Laboratory of Biotherapy and Cancer Center, West China Hospital, Sichuan University and Collaborative Innovation Center of Biotherapy, Chengdu 610041, China
* Corresponding authors

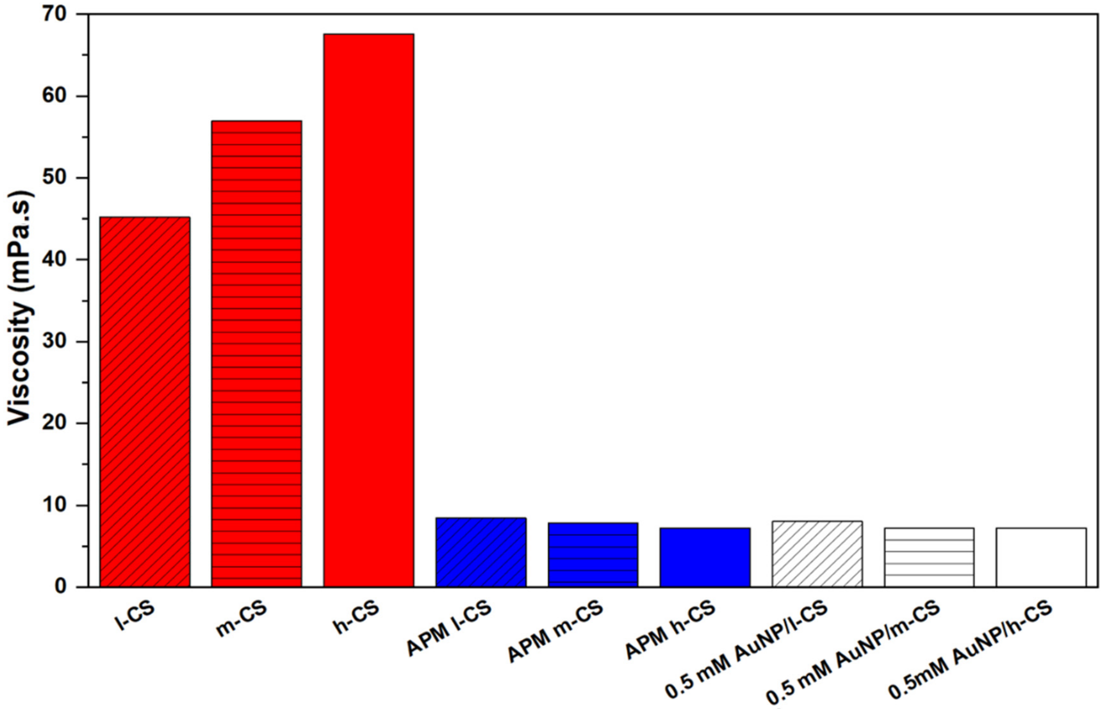


**Figure S1**. The viscosities of samples under investigation.

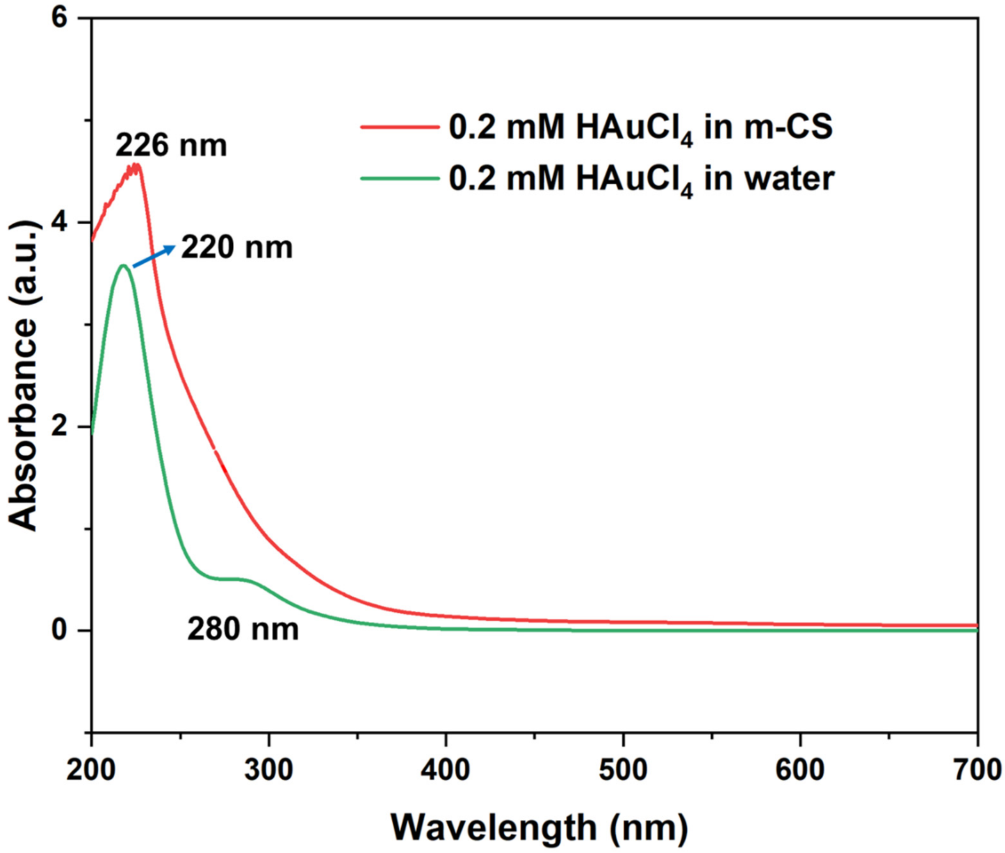


**Figure S2**. UV-vis spectra of 0.2 mM $HAuCl_4$/m-CS mixture and untreated 0.2 mM $HAuCl_4$ in water.

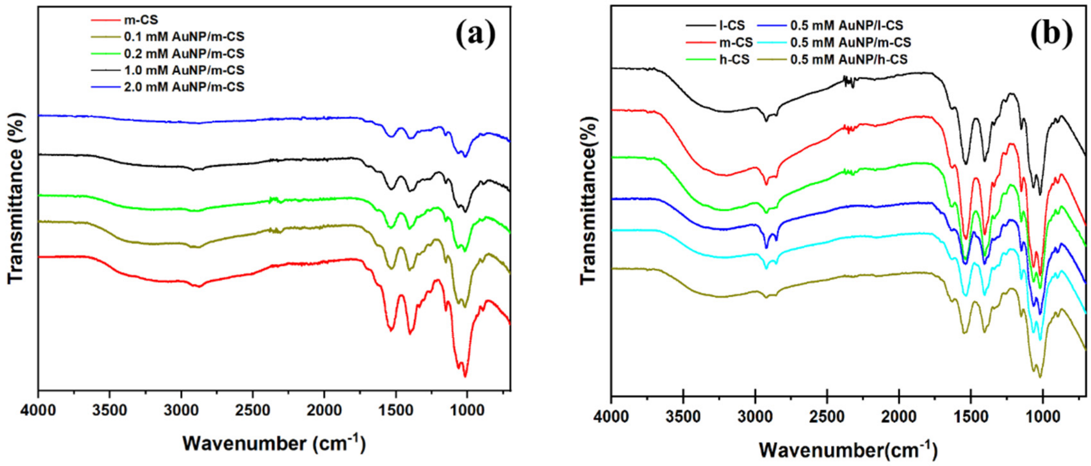


**Figure S3**. FTIR spectra of (**a**) untreated m-CS and APM-treated 0.1 mM AuNP/m-CS, 0.2 mM AuNP/m-CS, 1.0 mM AuNP/m-CS, and 2.0 mM AuNP/m-CS groups. (**b**) FTIR spectra of untreated (l, m, h)-CS and APM-treated 0.5 mM AuNP/(l, m, h)-CS groups.

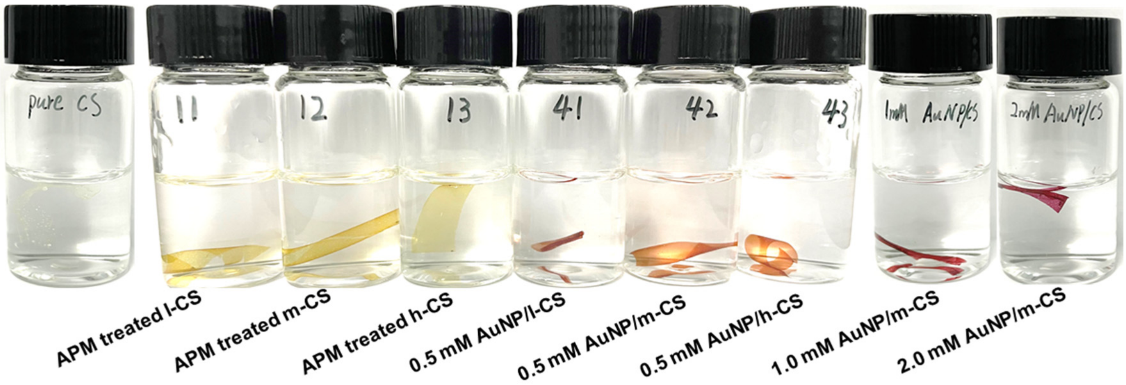


**Figure S4**. Image of sample films after immersed in PBS for 24 h at 37 °C.

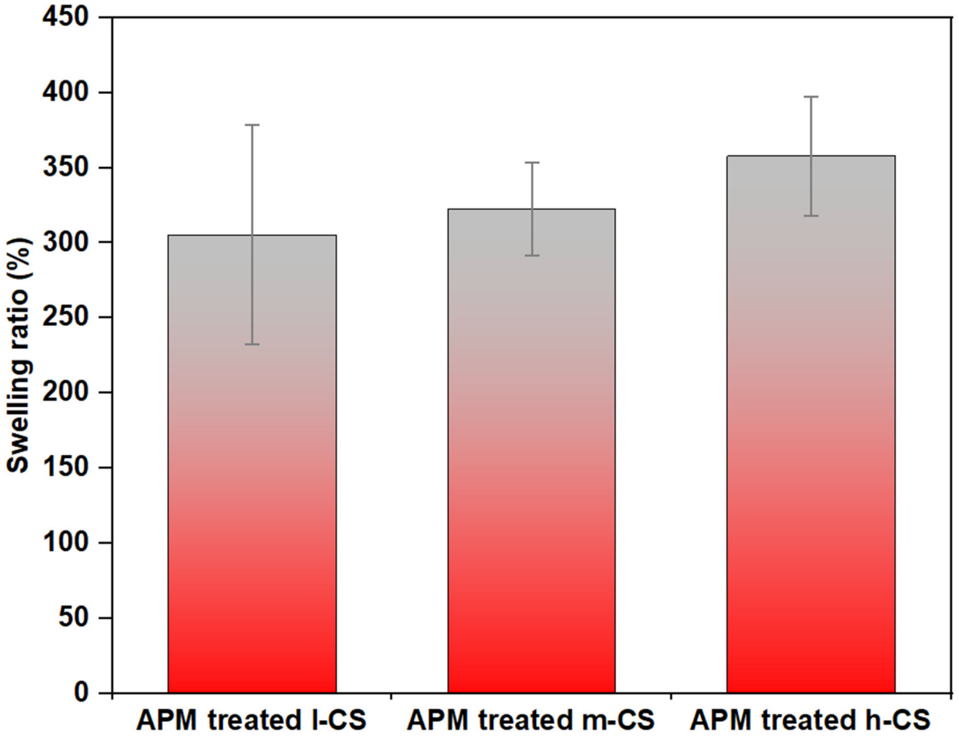


**Figure S5**. Swelling ratio of AMP-treated l-CS, m-CS and h-CS.

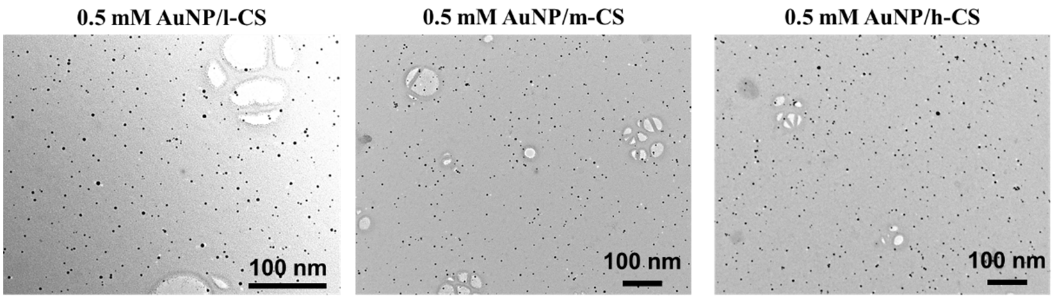


**Figure S6**. Typical low magnification TEM images of 0.5 mM AuNP/l-CS, 0.5 mM AuNP/m-CS, and 0.5 mM AuNP/h-CS nanocomposites.

**Table S1.** Au 4f peak information for 0.5 mM AuNP/l-CS, 0.5 mM AuNP/m-CS, and 0.5 mM AuNP/h-CS nanocomposites.

| sample | | Au species | | |
|---|---|---|---|---|
| | | $Au^0$ $4f_{5/2}$ | $Au^0$ $4f_{7/2}$ | $Au^{3+}$ |
| 0.5 mM AuNP/l-CS | BE (eV) | 87.80 | 84.07 | - |
| | Fraction (%) | 57.20 | 42.80 | - |
| 0.5 mM AuNP/m-CS | BE (eV) | 87.90 | 84.19 | - |
| | Fraction (%) | 44.56 | 55.54 | - |
| 0.5 mM AuNP/h-CS | BE (eV) | 88.82 | 84.56 | - |
| | Fraction (%) | 44.09 | 55.91 | - |
